# Functional and Immunological Studies Revealed a Second Superantigen Toxin in Staphylococcal Enterotoxin C Producing *Staphylococcus aureus* Strains

**DOI:** 10.3390/toxins14090595

**Published:** 2022-08-29

**Authors:** Andreas Roetzer, Nina Model, Jakob Laube, Yvonne Unterhumer, Guenter Haller, Martha M. Eibl

**Affiliations:** Biomedizinische Forschung & Bio-Produkte AG, 1090 Vienna, Austria

**Keywords:** *Staphylococcus aureus*, staphylococcal enterotoxins, mitogenesis, neutralisation

## Abstract

*Staphylococcus aureus* is a human and animal pathogen as well as a commensal bacterium. It can be a causative agent of severe, life-threatening infections with high mortality, e.g., toxic shock syndrome, septic shock, and multi-organ failure. *S. aureus* strains secrete a number of toxins. Exotoxins/enterotoxins are considered important in the pathogenesis of the above-mentioned conditions. Exotoxins, e.g., superantigen toxins, cause uncontrolled and polyclonal T cell activation and unregulated activation of inflammatory cytokines. Here we show the importance of genomic analysis of infectious strains in order to identify disease-causing exotoxins. Further, we show through functional analysis of superantigenic properties of staphylococcal exotoxins that even very small amounts of a putative superantigenic contaminant can have a significant mitogenic effect. The results show expression and production of two distinct staphylococcal exotoxins, SEC and SEL, in several strains from clinical isolates. Antibodies against both toxins are required to neutralise the superantigenic activity of staphylococcal supernatants and purified staphylococcal toxins.

## 1. Introduction

*Staphylococcus aureus* is a human and animal pathogen as well as a commensal bacterium. It colonises the skin and mucosal surfaces of up to one fourth of the human population at any given time [1,2]. However, it is also a major cause of life-threatening illnesses [3,4]. The versatile pathogenicity of *S. aureus* is based on its potential to express, produce, and secrete virulence factors such as exotoxins, thus interfering with the natural barriers of infections [5,6]. In general, Gram-positive bacterial, staphylococcal, streptococcal, and fungal exotoxins can have a similar impact on the immune cells of the host [7,8].

Staphylococcal superantigens are a diverse group of serologically distinct exotoxins including toxic shock syndrome toxin 1 (TSST-1), as well as several staphylococcal enterotoxins (SEs) (serotypes A-E and G-I) and numerous SE-like (SEL) superantigen toxins (serotypes K-T) [9,10]. To date, 32 serologically distinct staphylococcal superantigen toxins have been identified [6,10]. Staphylococcal enterotoxins (SEs) have been defined by their emetic activity. SE-like proteins are structurally similar to SEs but either lack emetic activity or have not yet been characterised for this function [9]. SEs have been shown to be causative agents in the pathogenesis of systemic infections as well as severe organ-specific diseases [5,6].

Superantigens can induce T cell and B cell lineages, and furthermore, induce innate immune response pathways to stimulate effector cells such as neutrophils and macrophages [11,12,13]. They bind to the major histocompatibility complex (MHC) and can interact with the antigen-specific region of the T cell receptor (TCR) via the variable region Vβ [14]. Superantigens can also be a cause to activate macrophages; they in turn activate effector functions and mononuclear cells (MNCs) to release modulators [11]. Stimulation of cytokine production through superantigens can also depend on various innate immune functions, e.g., via the activation of toll-like receptor pathways [15]. The induction of up to 20% of T cells and activation of macrophages can trigger a cytokine storm [16].

Superantigen toxins may cause systemic diseases such as toxic shock syndrome (TSS) [14]. The main toxins causing TSS are TSST-1, SEB, and SEC. Examples of organ-specific illnesses caused by superantigen toxins include toxic pneumonia, endocarditis, and atopic dermatitis [17,18,19]. Superantigen toxins can also affect the gastrointestinal tract. Additionally, staphylococcal enterotoxins and exotoxins have been suggested to play a pathogenic role in inflammatory bowel diseases [20]. 

During the development of a protective toxoid vaccine against SEC, we investigated the genetic, molecular, and functional properties of the toxin. SEC has been shown to cause TSS even in the absence of TSST-1 [21,22,23]. Furthermore, SEC has been associated with (haemorrhagic) pneumonia and purpura fulminans [22,24,25]. SEC subtypes also play a causative role in staphylococcal food poisoning [10,26]. 

Several superantigen toxins have been shown to be co-expressed and co-produced in a variety of combinations in *S. aureus*. In a previous study from our lab, the analysis of 51 strains of clinical isolates revealed that 11.8% (6 out of 51 strains) carried both the *sec* and the *sel* genes [27]. SEL was initially characterised as non-emetic in primates, while it showed emetic activity in rabbits [28]. More recent work has shown the emetic activity of SEL in further animal models, which suggests that this toxin should potentially be considered as a food safety hazard [29,30]. Both SEC and SEL were detected in contaminated food samples [31]. Genes encoding both proteins have additionally been found combined on pathogenicity islands in *S. epidermidis* [32,33].

In this study, we further characterised the supernatants secreted from staphylococcal strains mainly containing enterotoxin C. We found traces of a contaminant, which highly affected the superantigenic potential of SEC. Genomic analysis and protein characterisation using a polyclonal SEL antiserum proved that SEL was the second mitogen responsible for dysregulated T cell activation/dysregulated inflammation. Furthermore, we showed that six strains from fresh clinical isolates of two Austrian hospitals carry both the *sec* and *sel* gene. We demonstrated the genomic presence and co-expression of SEC and SEL and that each toxin alone was able to induce polyclonal T-cell activation and inflammation. Our results showed that antiserum raised against each of the toxins can neutralise the respective antigen in vitro. Antiserum raised against one toxin could not neutralise the other toxin. Due to the low amounts of SEL in our samples, its presence was determined by neutralisation measurements and semi-quantitative Western blot. Nevertheless, these low concentrations of SEL were able to induce T cell activation. Therefore, we want to highlight the necessity of multi-component vaccine development against *S. aureus* superantigen toxins.

## 2. Results

### 2.1. Purified SEC and SEL Stimulates Proliferation of MNCs 

In our previous study, we found six strains producing the superantigen toxin SEC and identified the genomic presence of the superantigen toxin SEL. While the presence of all known superantigens was genomically analysed, produced amounts of SEC in bacterial supernatants were quantified [27]. Other exotoxins suchas α-toxin were present and produced amounts of toxins varied. The overall genomic heterogeneity of strains was not found in *sec*-positive strains, all of which showed identical gene patterns.

Here, laboratory strain 19095 used in previous clinical studies was analysed and had a similar expression pattern: the strain secreted high amounts of SEC, but also other undefined toxins as demonstrated in different experiments (data not shown). Preliminary results from polymerase chain reaction screening and sequencing revealed the presence of the second superantigen gene *sel* in the bacterial strain [27].

**Figure 1:**
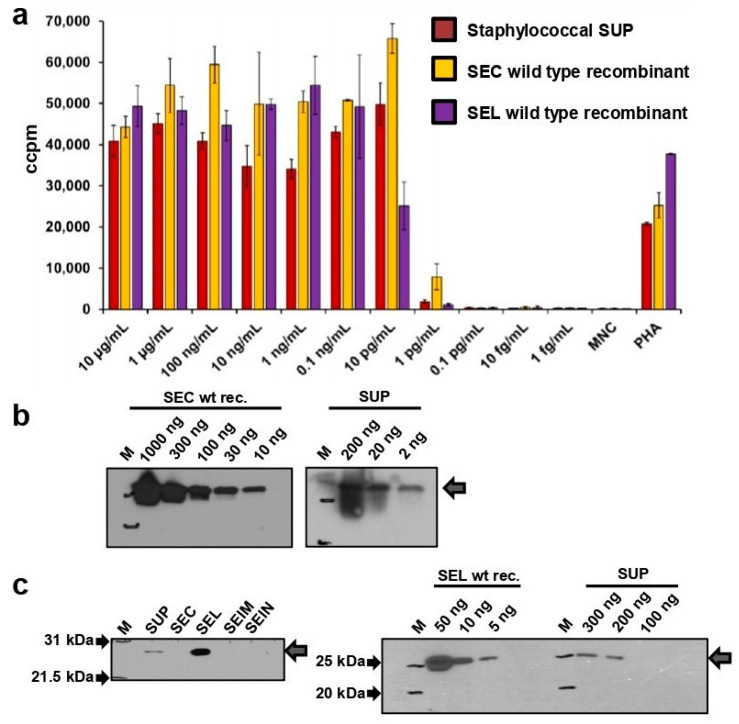
(**a**) Superantigen toxins have a broad range of MNC activation. Polyclonal T cell stimulation can be detected with toxin concentrations as low as 10 pg/mL. Proliferation of human peripheral blood MNCs stimulated with purified supernatant from *S. aureus* 19095 (SUP) and wild-type recombinant SEC and SEL. Incorporation of [^3^H] thymidine was counted. Experiments were carried out with MNCs from two different donors. Wild-type recombinant and native proteins were diluted in RPMI 1640 complete medium. MNCs were adjusted to 1 × 10^6^ cells per mL. Phytohaemagglutinin (PHA) was used as a positive control. (**b**) Estimation of SEC concentrations in supernatants of staphylococcal strain 19095. Well-defined amounts of recombinant SEC were compared to different amounts of supernatant of strain 19095. Anti-SEC antiserum was used for detection. (**c**) SEC and SEL are present in different concentrations in the supernatant of staphylococcal strain 19095. Estimation of SEL concentrations in purified supernatants of staphylococcal strain 19095, analysed by Western blot. An amount of 50 ng of bacterial supernatant of laboratory strain 19095 (SUP) or recombinant wild-type proteins (SEC, SEL, SElM, SElN) was loaded on a polyacrylamide gel, electrophoresed, and transferred to a nitrocellulose membrane. PageRuler^TM^ Plus (Biorad; left panel) and Precision Plus Protein^TM^ (Biorad; right panel) were added as ladder to identify SEL bands. Anti-SEL antiserum was used for detection of SEL.

Induction of uncontrolled T cell activation is an important indicator of superantigen activity. In order to test the specific mitogenic potential of the chromatographically purified staphylococcal supernatant of strain 19095 (SUP), human MNCs were treated with this supernatant or recombinant SEC and SEL produced in *E. coli*. The incorporation of [^3^H]-thymidine was measured to quantify the proliferation of MNCs. All experiments showed comparable stimulation of proliferation at doses as low as 10 pg/mL (Figure 1a). A lack of mitogenic activity was only observed at concentrations lower than 1 pg/mL.

### 2.2. Western Blot Confirms Contamination of SEC Containing Staphylococcal Supernatant with SEL

Semi-quantitative Western blots were performed to further analyse the chromatographically purified bacterial supernatant containing enterotoxins of highly similar size (SEC 27.6 kDa, SEL 24.7 kDa). To confirm the presence of SEC in this purified staphylococcal supernatant, we used different amounts of recombinant wild-type SEC as well as native staphylococcal toxin samples (i.e., bacterial supernatant) and probed it with anti-SEC antiserum in Western blot experiments (Figure 1b). Comparing the 20 ng band of the bacterial supernatant to the 30 ng and 10 ng bands of the recombinant wild-type SEC protein, the amount of expressed SEC in the staphylococcal supernatant was found to be fitting to the comparable amounts from the dilution row.

Thereafter, we analysed the presence of the known superantigen toxin SEL in the 19095 supernatant. The use of an anti-SEL antiserum demonstrated that signals appear in the lanes loaded with the staphylococcal supernatant and recombinant wild-type SEL (Figure 1c, left panel). Wild-type recombinant SEC, SE*l*M, and SE*l*N proteins were added as controls and did not produce any signal. This proved that SEL is a co-expressed, contaminating toxin in the supernatant of strain 19095. A semi-quantitative Western blot was performed, and we found that the signal of 5 ng recombinant wild-type SEL corresponded well to the 200 ng sample of the staphylococcal supernatant of 19095 (Figure 1c, right panel), resulting in approximately 2.5% SEL. Thus, this chromatographically purified supernatant consists of ≤97.5% SEC.

### 2.3. Neutralisation of Staphylococcal Supernatant and Recombinant SEC

Human MNCs from two different donors were incubated with 0.1 ng/mL of either staphylococcal supernatant or recombinant wild-type SEC. Increasing dilutions of antisera against staphylococcal supernatant or recombinant SEC were added to assess their potential to neutralise proliferation. A serum without specific antibodies was used as negative control. Incorporation of [^3^H] thymidine was measured to quantify the proliferation of MNCs. 

**Figure 2:**
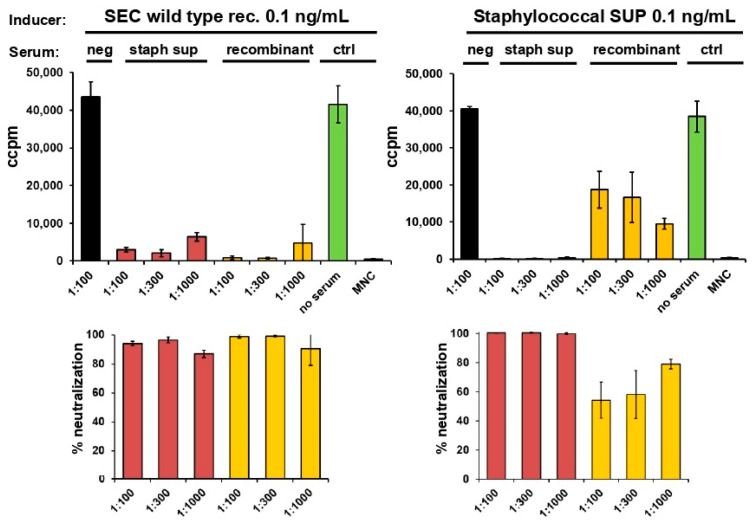
Anti-SEC antiserum alone cannot completely neutralise proliferation of MNCs induced by supernatant of staphylococcal laboratory strain 19095. Neutralisation of proliferation induced by recombinant SEC (left panel) or supernatants of staphylococcal strain 19095 (staph sup (SUP), right panel) is shown. Bars from antisera against purified staphylococcal supernatant are shown in red, bars from antisera against recombinant superantigen are shown in yellow, and bars of controls (no addition of sera, and MNCs only) are given in green. Proliferation of human peripheral blood MNCs was quantified by measuring [^3^H] thymidine incorporated. Experiments were conducted with MNCs from two independent donors. MNCs were adjusted to 1 × 10^6^ cells per mL. Percentage of neutralisation results (lower panels) were taken from means of triplicate values (tested in two donors) and normalised against the negative control (MNC).

Proliferation of MNCs incubated with the recombinant SEC could be effectively neutralised with sera against the chromatographically purified staphylococcal supernatant of the laboratory strain (19095) and recombinant wild-type SEC (Figure 2, left panel) with neutralisation efficiency over 80%. Proliferation of MNCs incubated with the staphylococcal supernatant (SUP) could only be effectively neutralised by the serum against the supernatant. The antiserum against the recombinant wild-type SEC showed incomplete neutralisation only (Figure 2, right panel). These results suggest that the staphylococcal supernatant contained not only SEC but was contaminated with an additional mitogenic substance. The genomic co-expression of the strain indicated this contamination to be SEL, therefore, antiserum against this superantigen was included in neutralisation experiments.

### 2.4. Neutralisation of Staphylococcal Supernatant and Recombinant SEC and SEL

**Figure 3:**
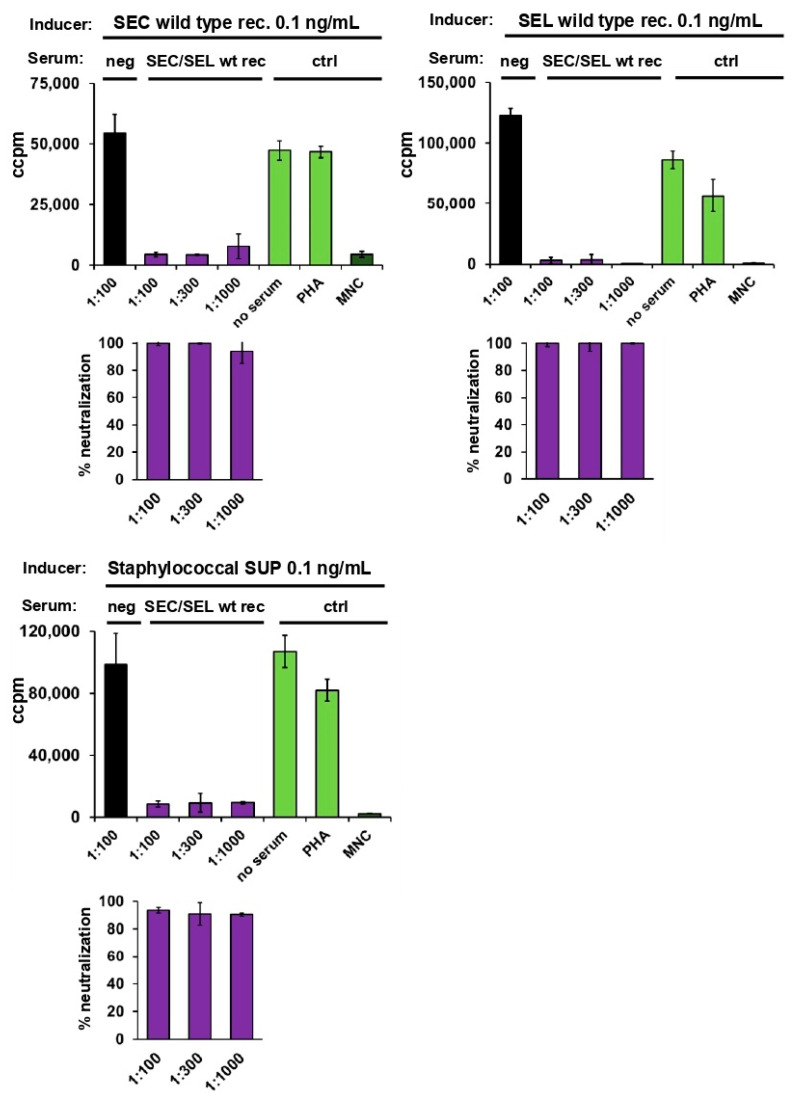
Both SEC and SEL antisera are required to neutralise proliferation of MNCs induced by the supernatant of staphylococcal laboratory strain 19095. Bars from antisera against recombinant superantigens SEC/SEL are shown in violet, and bars of controls are given in green (no addition of sera, PHA) and dark green (MNCs only). Proliferation of human peripheral blood MNCs was quantified by measuring [^3^H] thymidine incorporated. Experiments were conducted with MNCs from two independent donors. MNCs were adjusted to 1 × 10^6^ cells per mL. Percentage of neutralisation results (lower panels) were taken from means of triplicate values (tested in two donors) and normalised against the negative control (MNC).

Rabbits were immunised with a combination of recombinant SEC and SEL. The resulting serum was evaluated for its potential to neutralise recombinant SEC, recombinant SEL, as well as the staphylococcal supernatant (Figure 3). Hence, human MNCs were stimulated with 0.1 ng/mL staphylococcal supernatant (laboratory strain 19095), wild-type recombinant SEC, or wild-type recombinant SEL. 

Antiserum derived from rabbits immunised with the SEC and SEL mixture was added in increasing dilutions. The serum was able to neutralise proliferation of MNCs induced by both recombinant toxins alone (Figure 3, upper panel). Furthermore, the two-component antiserum effectively neutralised proliferation induced by the staphylococcal supernatant (Figure 3, lower panel). 

These results show that the staphylococcal supernatant indeed contained traces of SEL and that there was no additional mitogenic contaminant responsible for the strong proliferation of MNCs. For complete neutralisation of the mitogenic effects of the two superantigen toxins, immunisation with both SEC and SEL was necessary. These results clearly demonstrate that neutralisation of staphylococcal superantigens can be facilitated by genomic analysis of the responsible strains. Antibodies raised against recombinant SEC alone (4 times 30 µg) did not completely neutralise toxin-induced uncontrolled T cell activation. However, the combination of antibodies induced by recombinant SEC and SEL did.

### 2.5. Assessment of Antibody Titres against Wild-Type and Toxoid SEC by ELISA

To identify the antigenicity of the main toxins of interest, ELISAs were established to determine the antibody titres of the sera against the staphylococcal supernatant and the recombinant SEC. Rabbits were immunised with the bacterial supernatants as well as with wild-type recombinant SEC or SEC toxoid, all formulated with Al(OH)_3_. Al(OH)_3_ alone was used as a control. Plates were coated with either the staphylococcal supernatant of the laboratory strain or the recombinant wild-type SEC. Titres are shown on a logarithmic scale on the y-axis (Figure S1). All three sera displayed high antibody binding titres. These results confirmed that the obtained sera contained antibodies against the proteins of interest and could be used for the quantification of their toxin neutralising potential. Even the toxoid yielded high antibody titres, which is an important indication of immunogenicity in the toxoid vaccine development. Antiserum raised against the staphylococcal supernatant from the laboratory strain also neutralised polyclonal T cell activation, i.e., mitogenicity (see Figure 2), again proving the presence of residual contamination.

### 2.6. Proliferation of Mononuclear Cells Induced by Six Different Staphylococcal Strains Could Be Neutralised by a Combination of Antisera Raised against Single Superantigens

**Figure 4:**
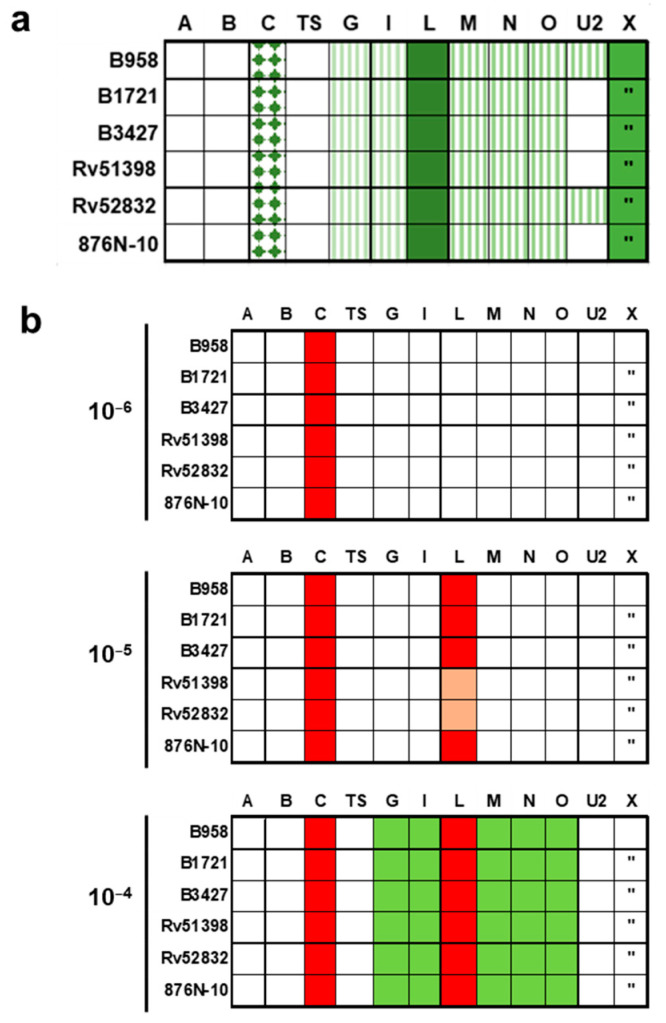
(**a**) Genomic superantigen repertoire of the six strains reported in this study. Sequencing results revealed that the six strains expressing SEC and SEL also contain the genes for the egc operon (*seg*, *sei*, *selm*, *seln*, *selo*, and *selu2*, lined boxes). Big letters indicate (prominent) superantigen genes tested (e.g., A = *sea*, X = *selx*). *Selu2* was the only version of *selu* genes present. Quotation marks indicate truncated version of *selx* (boxes shown in green; members of the egc operon are shown with same stripe pattern). Blood strains are indicated as B, colonising strains are indicated as Rv or N-10. (**b**) Neutralisation of superantigen containing supernatants of six different strains at different dilutions. Supernatant diluted 10^−5^ needs antisera against SEC and SEL to be neutralised (red boxes), although strains Rv51398 and Rv52832 show only weak proliferation without anti-SEL antiserum (rosy boxes, middle panel). At a supernatant dilution of 10^−4^, proliferation is only inhibited by adding antisera against SEC as well as SEL, G, I, M, N, and O (lower panel).

In a previous study, 51 strains of clinical staphylococcal isolates were genetically analysed [27]. Out of the 51 collected strains, 6 (11.8%) carried the sec gene, and we observed that all strains also carried the sel gene (Figure 4a). Prominent superantigens SEA, SEB, and TSST-1 were absent in all SEC-positive strains. Both superantigen proteins could also be detected in the bacterial supernatants. However, we found a difference of 1–2 log steps in mitogenic activity. SEL was found in lower concentrations than SEC, but its activity was detectable in all strains. SEL was found in both colonising and blood isolates (three isolates from each group). Sequencing of sec revealed two subtypes, SEC1 and SEC2, in our strains (Figure S2). B958 was the only strain carrying sec1. The overall sequence homology is about 97%. Sequencing of sel revealed an even higher homology (99.2%). 

When MNCs were treated with different dilutions of crude, sterile-filtered staphylococcal supernatants, superantigen-associated proliferation was induced (Figure 4b). Bacterial supernatants of all six staphylococcal strains studied showed mitogenic potential even when diluted up to 10^−6^ (see overview in Figure 4b). Boxes in colours represent the superantigens, which needed to be neutralised at the indicated dilutions of supernatants (10^−4^–10^−6^) to inhibit proliferation. 

At a supernatant dilution of 10^−6^ (Figure 4b, upper panel) antiserum against SEC was sufficient to inhibit proliferation of all six strains (red boxes). The anti-SEC antiserum neutralised both SEC subtypes to the same extent. Expression of *sel* in the six strains was detected at a supernatant dilution of 10^−5^ (red boxes). In detail, all six supernatants of strains showed SEC- and especially SEL-dependent neutralisation to different extents at a dilution of 10^−5^ (Figure 4b, middle panel). Colonising strains Rv51398 and Rv52832 displaying a lesser dependence on SEL antisera for neutralisation are indicated as light red boxes (Figure 4b, middle panel). At a dilution of 10^−4^, all six supernatants showed an SEC/L-dependent but also an SEG-, SEI-, SE*l*M-, SE*l*N-, and SE*l*O-dependent neutralisation, showing the involvement of the superantigens encoded on the egc operon at this supernatant dilution level (Figure 4b, lowest panel). Sera against SE*l*U2 did not play a role in neutralisation.

**Figure 5:**
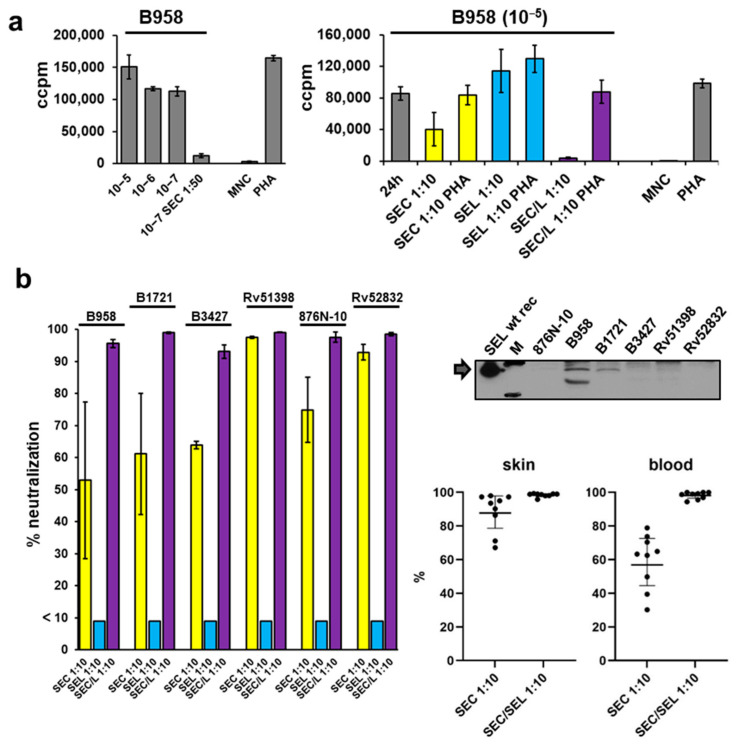
Proliferation of human peripheral blood mononuclear cells is stimulated by supernatants comprising the two superantigens SEC and SEL and can be neutralised by antisera against both. (**a**) Induced proliferation was measured by incorporation of [^3^H] thymidine. Each experiment was performed in triplicate. Bacterial strains were centrifuged and filtrated, cell-free supernatants including sub-cellular particles were analysed. Diluted supernatants were neutralised with antisera (x-axis annotation; bars for SEC neutralization are shown in yellow, SEL in blue, and SEC/SEL in violet) for 1 h at 37 °C (900 rpm). The mitogen PHA was added to neutralisations as control to reveal cytotoxic effects. (**b**) Percentages of neutralisation of supernatants. Different suspensions of antisera were applied in a 1:10 dilution to specific 10^−5^-fold diluted supernatants of tested SEC-producing strains. After incubation, mixes were added to isolated blood cells, and incorporation of [^3^H] thymidine was measured. Each experiment was performed in triplicate (left panel). Western blot analysis of collected bacterial samples with a polyclonal antiserum raised against SEL. PageRuler^TM^ Plus (Biorad) was added as ladder to identify correct bands (25 kDa and 15 kDa bands are shown on the blot). An amount of 50 ng SEL wild-type recombinant protein was used as positive control (right upper panel). Scattered dot plots with geometric means (including 95% CI) of percentages of supernatant neutralisations are shown. Three blood isolates and three colonising isolates tested in triplicates resulted in nine neutralisation values for both groups, which were included here (right lower panel).

In more detail, addition of anti-SEC antiserum at a dilution of 1:50 completely inhibited proliferation induced by the supernatant of strain B958 at a dilution of 10^−7^ (Figure 5a left panel). At a dilution of 10^−5^, the supernatant of strain B958 induced proliferation of human MNCs and addition of 1:10 diluted antisera against SEC or SEL alone was not sufficient to completely inhibit proliferation (Figure 5a, right panel). A combination of anti-SEC/SEL antisera diluted 1:10 was essential for neutralisation of proliferation. The high concentration of antisera (1:10) was chosen to ensure that SEC is fully neutralised and thus the remaining activity can be assigned to SEL. To exclude inhibitory effects of the highly concentrated antisera, phytohemagglutinin (PHA) was added to the experiments. 

MNCs were stimulated with 1:10^5^ diluted supernatants of all six SEC/SEL producing strains B958, B1721, B3427, Rv51398, 876N-10, and Rv52832 (Figure 5b, left panel). Antisera against SEC, SEL, or combined SEC/SEL at a dilution of 1:10 were added. Addition of anti-SEC antiserum alone could effectively inhibit proliferation induced by supernatants of colonising strains Rv51398 and Rv52832 but showed weaker neutralisation ability against strain 876-N10 and the blood strains B958, B1721, and B3427. Antisera against SEL did not show any effect due to the presence of mitogenic SEC (SEL bars are given as <10 instead of 0, without standard deviations). Only antisera against both SEC and SEL could effectively neutralise proliferation induced by supernatants of the latter. 

Additional Western blot experiments were performed to analyse the occurrence of SEL in the six selected supernatants. Strains that exhibited a visible band for SEL (B985, B1721; Figure 5b, upper right panel) were the same that required combined SEC and SEL antiserum to be neutralised. Samples from colonising strains did not lead to visible SEL signals in Western blot analysis. This corresponds to the results of the neutralisation experiments described above. 

Scattered dot plots with geometric means (including 95% confidence intervals (CI)) of percentages of supernatant neutralisations of the six strains analysed are shown (Figure 5b lower right panel). Three blood isolates and three colonising isolates tested in triplicates resulted in nine neutralisation values for both groups. In summary, data from skin strains showed a lesser dependence on SEL neutralisation than blood isolates (skin *p* values 0.03, blood *p* values < 0.0001). However, the significance of these results is limited due to the low number of strains tested.

In the presented study, we show activation of human MNCs by a model laboratory strain and six bacterial isolates collected in two Austrian hospitals. The results of the experiments in this study demonstrate expression of two staphylococcal toxins (SEC and SEL) in the analysed strains and revealed an MNC activation, which could not be neutralised with one antiserum alone.

## 3. Discussion

Superantigen toxins described in this study have various dramatic effects on the immune system. They lead to uncontrolled inflammation in a broad range of concentrations and activate innate immune cells. In a previous study, we described genotypic and phenotypic analyses of 51 staphylococcal strains collected in two Austrian general hospitals in Vienna and Linz [27]. In the previous study, SEL could not be detected by ELISA. In this study, we focused on the genotypic and phenotypic analysis of *S. aureus* strains co-expressing SEC and additional superantigens, in this case SEL. In a large number of literature references, we found one publication describing the co-expression of SEC and SEL [16]. As a model, *S. aureus* laboratory strain 19095, expressing SEC and SEL, was chosen. All strains were tested to be methicillin-sensitive *S. aureus* (MSSA); genomic sequencing data were used for the identification of genes coding for staphylococcal exotoxins [27]. Expression of the respective proteins was examined by Western blotting. Further, we performed functional immunological analyses of secreted toxins. 

For the detection of SEL, a highly sensitive semi-quantitative Western blot was used, which was able to detect SEL in a concentration of 2–5% of secreted SEC. Among all 51 collected strains, 6 (11.8%) carried the *sec* gene and all these 6 strains also carried the gene encoding for SEL. The six strains were equally distributed among colonising and blood isolates (three isolates in each group). The percentage of SEC/SEL co-expressing strains in blood isolates was slightly but not significantly higher (13.6%, 3 out of 22) than the percentage of colonising strains (10.3%, 3 out of 29). 

Proliferation of MNCs induced by recombinant SEC could be neutralised by antiserum against recombinant SEC derived from rabbits as well as by antiserum against the isolated and purified staphylococcal toxin from bacterial supernatants. Using this staphylococcal toxin, proliferation could only be effectively neutralised by antiserum against itself, while antiserum against recombinant SEC showed incomplete neutralisation only. This led us to suspect that the native SEC contained minute amounts of another mitogenic substance. Based on the genomic analysis, we suspected that SEL would be the most likely contaminant. Applying antibodies against both SEC and SEL raised by immunisation with the recombinant toxins inhibited superantigenic activity of staphylococcal toxin effectively. 

By semi-quantitative Western blot, it was possible to detect very low amounts of SEL in the supernatants of two staphylococcal strains, as well as in the staphylococcal toxin isolate which was taken from bacterial supernatant. These results highlight the importance of genomic analysis of the fresh clinical isolates to facilitate recognition of a contaminant superantigen toxin. A high background level due to the antibody Fc-domain (fragment crystallisable) binding capacity of *S. aureus* supernatants can often affect blotting results of staphylococcal supernatants [27]. Hence, traces of secreted SEL from the colonising strains are likely to be undetectable using this technique. 

The pan-genome of *S. aureus* encodes several superantigen toxins. Most of these genes are located on mobile genetic elements, resulting in a non-random heterogeneity in the configuration of these toxin genes in individual strains. SEC and SEL are often found to be co-localised on several pathogen-associated genetic elements [34,35,36]. SEC and SEL were also found in combination in the six strains tested here, underlining the presumed importance of SEL in SEC-producing blood isolates. Thus, we focused on the SEC–SEL interdependence and its implication. SEC1 and SEC2 subtypes showed no differences in proliferation, indicating the lack of importance of these genetic differences or mutations for superantigenic activity.

However, genomic analysis revealed the presence of other staphylococcal enterotoxin encoding genes and staphylococcal enterotoxin-like encoding genes. The gene cluster egc, designated as enterotoxic gene cluster, a highly prevalent operon of enterotoxin genes, was found in every SEC-producing strain [37]. Four out of six isolated strains carried the egc cluster consisting of five genes, while two strains carried the egc cluster plus *selu2*, a version of *selu* [38]. This high homogeneity was not found in other determined *S. aureus* groups: for example, *tst*-bearing isolates showed several other gene combinations [27]. 

The production of egc proteins among the analysed isolates was previously determined, revealing very low amounts of secreted proteins [37]. The importance of SEL was proven in a serial dilution of staphylococcal supernatants. At a 10^5^ dilution, it was essential to neutralise both SEC and SEL. A ten-fold lower dilution was necessary to see a mitogenic effect of the egc operon (data not shown). Therefore, we focused our efforts to determine the role of SEL. Using genomic analysis and improved methodology including semi-quantitative Western blots and neutralisation experiments, we identified SEL as a kind of contaminant toxin, thereby contradicting our previous observation of the lack of genotype/phenotype correlation in superantigen toxins in these strains. 

Looking at the effects of superantigen toxins on human MNCs, it became clear that the effect of activation of cells of the human monocyte macrophage lineage, inducing dysregulated T cell activation and inflammation (TNFα, IFNγ), occurred very fast. This effect was reminiscent of a previous experience where we studied the effects on induction of the cytokine storm [39]. The toxin induces a panic reaction: most likely, the first step involves a primary cell of innate immunity of the monocyte macrophage lineage that induces, if exposed to very low concentrations of the toxin, a polyclonal T cell activation and uncontrolled inflammatory response [11,40]. Assessing the properties of antisera raised against the toxin and the respective recombinant proteins clearly proved the functional difference and led the way to identify minute amounts of the contaminant. It was definitely surprising that such low amounts of the SEL toxin present in staphylococcal supernatants could induce both, binding and neutralising antibodies with similar kinetics and potential. 

Ongoing projects try to understand the mechanism more closely. In the small numbers of clinical isolates used in the presented study (three colonising and three systemic), we identified the contaminant with sensitive methods. Semi-quantitative determinations of the toxins in different experimental settings were performed. Numerous further experiments with well-defined bacterial strains are necessary to understand the details of the observed, possibly higher production of the contaminant in systemic isolates.

Staphylococcal exotoxins are responsible for a variety of diseases such as TSS and sepsis and severe inflammatory disease in single organs, e.g., toxic pneumonia and gastrointestinal diseases including food poisoning [14,41,42]. Therefore, we consider it of great importance to develop multi-component vaccines against staphylococcal exotoxins [43].

The presented results are pointing out the importance of genomic analysis of infective strains and functional studies of staphylococcal exotoxins to bring us a step closer to understanding the induction of a cytokine storm observed as a life-threatening complication of bacterial sepsis [44,45]. 

## 4. Materials and Methods 

### 4.1. Isolation of Supernatants

SEC-expressing staphylococcal cultures, growth conditions, and primer sequences were described in a previous report [27]. In short, for the isolation of secreted superantigen toxins from bacterial supernatants, collected *S. aureus* strains were cultured to stationary phase in 25 mL of tryptic soy broth at 37 °C with shaking at 170 revolutions per minute. The cultures were centrifuged at 3220× *g* for 5 min at indicated time points, followed by a sterile filtration of supernatants (PALL Acrodisc 25 mm Syringe Filters with 0.2 µm Posidyne Membrane). Protein samples were stored at −20 °C.

### 4.2. Purification of Native Staphylococcal SEC

Wild-type native staphylococcal toxin (putative SEC) was isolated from *S. aureus* laboratory strain 19095. Supernatants with secreted toxin were suspended with SP Sepharose FF, and pH was adjusted with 2M citrate buffer (to pH 5.5) and loaded on an XK 50/30 column with a 25 mM citrate buffer (pH 5.5). Proteins were eluted with 25 mM citrate buffer (pH 5.5) and sodium chloride buffer (pH 5.5). The peak fraction was adjusted with 2 M citrate buffer (pH 5) and 1.5 M ammonium sulphate and applied to HIC columns (GE Healthcare). Proteins were eluted with 25 mM citrate buffer (pH 5), peak fractions were applied to a HiPrep 26/10 desalting column, and proteins were eluted with 25 mM Tris buffer (pH 7.2). Peak fractions were applied to a HiTrap Capto Q column, and proteins were eluted with 25 mM Tris buffer (pH 7.2). The peak fraction was dialysed against 1X PBS and samples were stored at −20 °C. 

### 4.3. Expression and Purification of Recombinant Proteins

SEC wild-type gene was detoxified by mutating MHCII and TCR binding sites, resulting in a non-toxic SEC mutant protein. *S. aureus* superantigen wild-type toxins SEC and SEL and SEC toxoid were cloned, expressed, and purified in our facilities. Briefly, *Escherichia coli* strains (One Shot BL21-AI, Invitrogen, Carlsbad, CA, USA) transformed with a pET expression vector (Novagen, Madison, WI, USA) carrying cloned superantigen genes were grown at 28 °C and protein expression was induced by arabinose for 24 h. The pellets from protein-expressing *E. coli* bacteria containing the recombinant superantigen SEC was resuspended in 25 mM citrate buffer (pH 5.5), and the pellet containing the recombinant superantigen SEL was resuspended in phosphate buffer (pH 8), followed by sonication. Suspensions were loaded on SP-Sepharose FF columns (GE Healthcare, Little Chalfont, UK), and proteins were eluted with a 1M NaCl gradient. The peak fraction was dialysed against Tris (pH 8) and applied to Q-Sepharose FF columns (GE Healthcare). The peak fraction was dialysed against 1X PBS and samples were stored at −20 °C. Quantifications of all protein purifications were conducted using Bradford (OZ Biosciences, Marseille, France).

### 4.4. Western Blot Analyses

Western blots were performed as previously described [27]. Antisera used as primary antibodies against supernatant were used in a 1:40,000 dilution (1X TBS-T (with 1% Tween 20) and 5% milk powder (Roth, Karlsruhe, Germany)), and secondary antibodies were diluted 1:50,000 (1X TBS-T). Antisera used as primary antibodies against isolated proteins were employed in a 1:30,000 dilution (1X TBS-T and 5% milk powder), and secondary antibodies were diluted 1:30,000 (1X TBS-T). 

### 4.5. Lymphocyte Proliferation Assay

Peripheral blood mononuclear cells (PBMC) from healthy donors were isolated, adjusted, and cultured in complete RPMI 1640 medium as described in [46]. Addition of supernatants, incubation of cells, and analysis of incorporated radioactivity was carried out as previously described [27]. Each experiment was performed in triplicates.

### 4.6. Immunisation of Rabbits

To produce antisera, female New Zealand white rabbits (1.5–2 kg) were purchased from Charles River Laboratories (Sulzfeld, Germany). Animals were kept in standard care facilities according to the guidelines of the Austrian Ministry for Science and Research and had free access to food (Altromin 2120 standard diet pellets: Marek Futtermittelwerke, Vienna, Austria) and water. The animal experiments were approved and controlled by the Veterinary Department of the City of Vienna (M58/001295/2011/8, on 27 April 2011).

Animals were immunised four times in a three-week interval with 30 µg SEC wild-type native toxin or 30 µg wild-type recombinant toxin (SEC or SEL) or 30 µg recombinant SEC toxoid, all formulated with 1 mg AL(OH)_3_ in phosphate-buffered saline (PBS); 1 mg AL(OH)_3_ formulated in PBS was used as control. Blood was drawn before each immunisation and sera was obtained by centrifugation at 1500× *g* at room temperature. The sera were stored at −20 °C for further analysis.

### 4.7. Enzyme-Linked Immunosorbent Assay (ELISA)

ELISA to determine antibody titres in this lab were described before [37]. Flat bottom high-adsorbent 96-well plates were coated with 50 µL either recombinant SEC wild-type or native SEC wild-type protein in sodium carbonate buffer (pH 9.6) at a concentration of 0.12 µg/mL per well. After overnight incubation at 4 °C, the plates were washed four times with 400 µL/well washing buffer (1 × PBS, 0.1% polysorbate 20), blocked with 200 µL/well blocking buffer (1 × PBS, 3% BSA), and incubated at 37 °C for one hour. After incubation the plates were frozen at −20 °C until use. All sera and controls were pre-diluted in sample buffer (1 × PBS, 3% BSA, 0.1% polysorbate 20). Thawed plates were washed with 400 µL/well washing buffer and tapped over kitchen roll paper. An amount of 100 µL sample buffer was filled in each well except the first row of the plate, 200 µL of the pre-diluted samples and controls were filled in the first row of the plate, and a three-fold dilution was performed. The plates were incubated for one hour at 37 °C, washed with 400 µL/well washing buffer, and tapped over kitchen roll paper. A horseradish peroxidase-labelled anti-rabbit immunoglobulin G (IgG) antibody was diluted 1:25,000 in sample buffer and 50 µL/well was added. The plates were incubated for one hour at 37 °C, washed with 400 µL/well washing buffer, and tapped over kitchen roll paper. An o-phenylenediamine dihydrochloride tablet (OPD) was dissolved in substrate buffer (0.5 M phosphate-citrate-buffer) and 20 µL of a 30% H_2_O_2_ solution was added. An amount of 100 µL substrate was added to each well and the plates were incubated at room temperature in the dark for 15 min; 100 µL 1% sulphuric acid solution was used to stop the colorimetric reaction. The optical density was determined at 492 nm and titres were calculated according to a four-parameter analysis with x = 0.5.

### 4.8. Neutralisation

Human PBMCs were isolated as described [46]. An amount of 100 µL of 1 × 10^6^ cells/mL was cultured in 96-well flat-bottom tissue culture plates with complete medium (RPMI 1640 with stable glutamine), 10% foetal calf serum (Hyclone, Logan, UK), 100 U/mL penicillin, and 100 U/mL streptomycin (Gibco, Grand Island, NY, USA) for one hour at 37 °C in a humidified atmosphere. Then, 200 µL containing 0.4 µg antigen (SEC wild-type recombinant; SEL wild-type recombinant; SEC wild-type native) was incubated with 200 µL of antisera in different dilutions (1:25; 1:75; 1:250) (anti-TSST-1; anti SEC/SEL; negative serum without specific antibodies) for one hour at 37 °C at 900 rpm. All antigen and sera were prepared in culture medium. Subsequently, 100 µL of the pre-incubated antigen–antisera combination or 100 µL of controls (medium, PHA 1:160, and 0.2 µg antigen without serum) were added to the cells. Cells were incubated as described above. Incorporated radioactivity was measured by a MicroBeta Trilux 1450 scintillation counter (Wallac, Turku, Finland). Proliferative response was indicated as ccpm.

### 4.9. Statistics

Between-group comparisons of blood and skin isolates were performed using the Welch’s *t*-test. All tests were two-tailed and a *p* value less than 0.05 was considered significant.

## Data Availability

Not applicable.

## Supplementary Materials

The following supporting information can be downloaded at: https://www.mdpi.com/article/10.3390/toxins14090595/s1. Figure S1. Binding antibody titres of polyclonal antibodies from rabbits immunised with staphylococcal supernatant (staph sup) isolated from *S. aureus*, recombinant wild-type SEC, and an SEC toxoid. Coating of 96-well plates was performed with staphylococcal supernatant (staph sup) and recombinant SEC. Titres are given in a logarithmic scale on the y-axis. Flat-bottom 96-well plates were coated with either recombinant SEC wild-type protein or staphylococcal supernatant (staph sup) at a concentration of 0.12 µg/mL. All sera and controls were pre-diluted in sample buffer. A horseradish-peroxidase-labelled polyclonal anti-rabbit IgG antibody was diluted 1:25,000 in sample buffer. The plates were incubated for one hour at 37 °C. An OPD tablet was dissolved in substrate buffer and a 30% H_2_O_2_ solution was added. After adding the substrate, the plates were incubated for 15 min in the dark at room temperature. A stop solution (1% sulfuric acid solution) was added to stop the colorimetric reaction. The plates were read for optical density at 492 nm using a plate reader and titres were calculated according to a four-parameter analysis with x = 0.5. Figure S2. Alignment of sequences of *sec* and *sel*. Alignments were conducted using the software ClustalW with default settings.
