# Supplementary material for: Functional and Immunological Studies Revealed a Second Superantigen Toxin in Staphylococcal Enterotoxin C Producing Staphylococcus aureus Strains
Source: Toxins (Basel). 2022 Aug 29;14(9):595. doi: 10.3390/toxins14090595 (PMC9504012; doi:10.3390/toxins14090595)
Supplement: Supplementary file 1 [file toxins-14-00595-s001.zip › toxins-1816431-supplementary.pdf]

Supplementary Materials: Functional and Immunological Studies Revealed a Second Superantigen Toxin in Staphylococcal Enterotoxin C Producing Staphylococcus aureus Strains

Andreas Roetzer, Nina Model, Jakob Laube, Yvonne Unterhumer, Guenter Haller and Martha M. Eibl

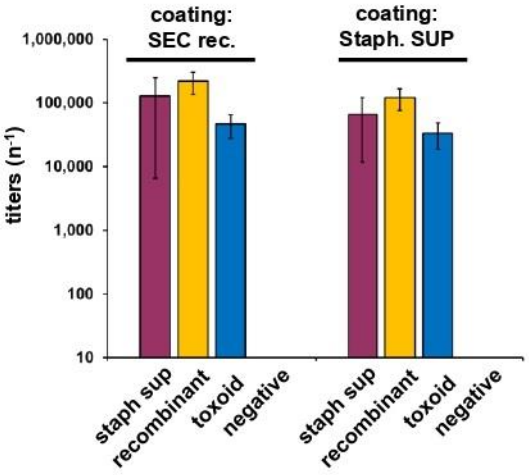

Figure S1. Binding antibody titres of polyclonal antibodies from rabbits immunised with staphylococcal supernatant (staph sup) isolated from S. aureus, recombinant wild-type SEC, and an SEC toxoid. Coating of 96-well plates was performed with staphylococcal supernatant (staph sup) and recombinant SEC. Titres are given in a logarithmic scale on the y-axis. Flat-bottom 96-well plates were coated with either recombinant SEC wild-type protein or staphylococcal supernatant (staph sup) at a concentration of 0.12 µg/mL. All sera and controls were pre-diluted in sample buffer. A horseradish-peroxidase-labelled polyclonal anti-rabbit IgG antibody was diluted 1:25,000 in sample buffer. The plates were incubated for one hour at 37 °C. An OPD tablet was dissolved in substrate buffer and a 30% H2O2 solution was added. After adding the substrate, the plates were incubated for 15 min in the dark at room temperature. A stop solution (1% sulfuric acid solution) was added to stop the colorimetric reaction. The plates were read for optical density at 492 nm using a plate reader and titres were calculated according to a four-parameter analysis with x = 0.5.

SEC alignment

```
SEC 876N-10    MNKSRFISCVILIFALILVLFTPNVLAESQPDPTPDELHKSSEFTGTMGNMKYLYDDHYV
SEC 52832      MNKSRFISCVILIFALILVLFTPNVLAESQPDPTPDELHKSSEFTGTMGNMKYLYDDHYV
SEC B1721      MNKSRFISCVILIFALILVLFTPNVLAESQPDPTPDELHKSSEFTGTMGNMKYLYDDHYV
SEC B3427      MNKSRFISCVILIFALILVLFTPNVLAESQPDPTPDELHKSSEFTGTMGNMKYLYDDHYV
SEC 51398      MNKSRFISCVILIFALILVLFTPNVLAESQPDPTPDELHKSSEFTGTMGNMKYLYDDHYV
SEC B958       MNKSRFISCVILIFALILVLFTPNVLAESQPDPTPDELHKASKFTGLMENMKVLYDDHYV
               ****************************************:*:*** * *** *******

SEC 876N-10    SATKVMSVDKFLAHDLIYNISDKKLKNYDKVKTELLNEDLAKKYKDEVVDVYGSNYYVNC
SEC 52832      SATKVMSVDKFLAHDLIYNISDKKLKNYDKVKTELLNEDLAKKYKDEVVDVYGSNYYVNC
SEC B1721      SATKVMSVDKFLAHDLIYNISDKKLKNYDKVKTELLNEDLAKKYKDEVVDVYGSNYYVNC
SEC B3427      SATKVMSVDKFLAHDLIYNISDKKLKNYDKVKTELLNEDLAKKYKDEVVDVYGSNYYVNC
SEC 51398      SATKVMSVDKFLAHDLIYNISDKKLKNYDKVKTELLNEDLAKKYKDEVVDVYGSNYYVNC
SEC B958       SATKVKSVDKFLAHDLIYNISDKKLKNYDKVKTELLNEGLAKKYKDEVVDVYGSNYYVNC
               ***** ********************************.*********************

SEC 876N-10    YFSSKDNVGKVTGGKTCMYGGITKHEGNHFDNGNLQNVLIRVYENKRNTISFEVQTDKKS
SEC 52832      YFSSKDNVGKVTGGKTCMYGGITKHEGNHFDNGNLQNVLIRVYENKRNTISFEVQTDKKS
SEC B1721      YFSSKDNVGKVTGGKTCMYGGITKHEGNHFDNGNLQNVLIRVYENKRNTISFEVQTDKKS
SEC B3427      YFSSKDNVGKVTGGKTCMYGGITKHEGNHFDNGNLQNVLIRVYENKRNTISFEVQTDKKS
SEC 51398      YFSSKDNVGKVTGGKTCMYGGITKHEGNHFDNGNLQNVLIRVYENKRNTISFEVQTDKKS
SEC B958       YFSSKDNVGKVTGGKTCMYGGITKHEGNHFDNGNLQNVLIRVYENKRNTISFEVQTDKKS
               ************************************************************

SEC 876N-10    VTAQELDIKARNFLINKKNLYEFNSSPYETGYIKFIENNGNTFWYDMMPAPGDKFDQSKY
SEC 52832      VTAQELDIKARNFLINKKNLYEFNSSPYETGYIKFIENNGNTFWYDMMPAPGDKFDQSKY
SEC B1721      VTAQELDIKARNFLINKKNLYEFNSSPYETGYIKFIENNGNTFWYDMMPAPGDKFDQSKY
SEC B3427      VTAQELDIKARNFLINKKNLYEFNSSPYETGYIKFIENNGNTFWYDMMPAPGDKFDQSKY
SEC 51398      VTAQELDIKARNFLINKKNLYEFNSSPYETGYIKFIENNGNTFWYDMMPAPGDKFDQSKY
SEC B958       ATAQELDIKARNFLINKKNLYEFNSSPYETGYIKFIENNGNTFWYDMMPAPGDKFDQSKY
               .***********************************************************

SEC 876N-10    LMMYNDNKTVDSKSVKIEVHLTTKNG
SEC 52832      LMMYNDNKTVDSKSVKIEVHLTTKNG
SEC B1721      LMMYNDNKTVDSKSVKIEVHLTTKNG
SEC B3427      LMMYNDNKTVDSKSVKIEVHLTTKNG
SEC 51398      LMMYNDNKTVDSKSVKIEVHLTTKNG
SEC B958       LMMYNDNKTVDSKSVKIEVHLTTKNG
               **************************
```

SEL alignment

```
SEL Rv51398     MKKRLLFVIVITLFIFSSNHTVLSNGDVGTGNLRNFYTKYEYVNLKNVKDKNSPESHRLE
SEL Rv52832     MKKRLLFVIVITLFIFSSNHTVLSNGDVGTGNLRNFYTKYEYVNLKNVKDKNSPESHRLE
SEL B3427       MKKRLLFVIVITLFIFSSNHTVLSNGDVGTGNLRNFYTKYEYVNLKNVKDKNSPESHRLE
SEL B1721       MKKRLLFVIVITLFIFSSNHTVLSNGDVGTGNLRNFYTKYEYVNLKNVKDKNSPESHRLE
SEL 876N-10     MKKRLLFVIVITLFIFSSNHTVLSNGDVGTGNLRNFYTKYEYVNLKNVKDKNSPESHRLE
SEL B958        MKKRLLFVIVITLFIFSSNHTVLSNGDVGPGNLRNFYTKYEYVNLKNVKDKNSPESHRLE
                *****************************.******************************

SEL Rv51398     YSYKNDTLYAEFDNEYITSDLKGKNVDVFGISYKYGSNSRTIYGGVTKAENNKLDSPRII
SEL Rv52832     YSYKNDTLYAEFDNEYITSDLKGKNVDVFGISYKYGSNSRTIYGGVTKAENNKLDSPRII
SEL B3427       YSYKNDTLYAEFDNEYITSDLKGKNVDVFGISYKYGSNSRTIYGGVTKAENNKLDSPRII
SEL B1721       YSYKNDTLYAEFDNEYITSDLKGKNVDVFGISYKYGSNSRTIYGGVTKAENNKLDSPRII
SEL 876N-10     YSYKNDTLYAEFDNEYITSDLKGKNVDVFGISYKYGSNSRTIYGGVTKAENNKLDSPRII
SEL B958        YSYKNDTLYAEFDNEYITSDLKGKNVDVFGISYKYGSNSRTIYGGVTKAENNKLDSPRII
                ************************************************************

SEL Rv51398     PINLIINGKHQTVTTKSVSTDKKMVTAQEIDVKLRKYLQDEFNIYGHNDTGKGKEYGTSS
SEL Rv52832     PINLIINGKHQTVTTKSVSTDKKMVTAQEIDVKLRKYLQDEFNIYGHNDTGKGKEYGTSS
SEL B3427       PINLIINGKHQTVTTKSVSTDKKMVTAQEIDVKLRKYLQDEFNIYGHNDTGKGKEYGTSS
SEL B1721       PINLIINGKHQTVTTKSVSTDKKMVTAQEIDVKLRKYLQDEFNIYGHNDTGKGKEYGTSS
SEL 876N-10     PINLIINGKHQTVTTKSVSTDKKMVTAQEIDVKLRKYLQDEFNIYGHNDTGKGKEYGTSS
SEL B958        PINLIINGKHQTVTTKSVSTDKKMVTAQEIDVKLRKYLQDEFNIYGHNDTGKGKEYGTSS
                ************************************************************

SEL Rv51398     KFYSGFDKGSVVFHMNDGSNFSYDLFYTGYGLPESFLKIYKDNKTVGSTQFHLDVEISKR
SEL Rv52832     KFYSGFDKGSVVFHMNDGSNFSYDLFYTGYGLPESFLKIYKDNKTVGSTQFHLDVEISKR
SEL B3427       KFYSGFDKGSVVFHMNDGSNFSYDLFYTGYGLPESFLKIYKDNKTVGSTQFHLDVEISKR
SEL B1721       KFYSGFDKGSVVFHMNDGSNFSYDLFYTGYGLPESFLKIYKDNKTVGSTQFHLDVEISKR
SEL 876N-10     KFYSGFDKGSVVFHMNDGSNFSYDLFYTGYGLPESFLKIYKDNKTVGSTQFHLDVEISKR
SEL B958        KFYSGFDKGSVVFHMNDGSNFSYDLFYTGYGLPESFLKIYKDNKTVDSTQFHLDVEISKR
                **********************************************.*************
```

Figure S2. Alignment of sequences of sec and sel. Alignments were conducted using the software ClustalW with default settings.
